# The Interplay Between Adipose Tissue and Vasculature: Role of Oxidative Stress in Obesity

**DOI:** 10.3389/fcvm.2021.650214

**Published:** 2021-03-04

**Authors:** Yawen Zhou, Huige Li, Ning Xia

**Affiliations:** Department of Pharmacology, Johannes Gutenberg University Medical Center, Mainz, Germany

**Keywords:** reactive oxygen species, adipokines, antioxidant, perivascular adipose tissue, vascular dysfunction

## Abstract

Cardiovascular diseases (CVDs) rank the leading cause of morbidity and mortality globally. Obesity and its related metabolic syndrome are well-established risk factors for CVDs. Therefore, understanding the pathophysiological role of adipose tissues is of great importance in maintaining cardiovascular health. Oxidative stress, characterized by excessive formation of reactive oxygen species, is a common cellular stress shared by obesity and CVDs. While plenty of literatures have illustrated the vascular oxidative stress, very few have discussed the impact of oxidative stress in adipose tissues. Adipose tissues can communicate with vascular systems, in an endocrine and paracrine manner, through secreting several adipocytokines, which is largely dysregulated in obesity. The aim of this review is to summarize current understanding of the relationship between oxidative stress in obesity and vascular endothelial dysfunction. In this review, we briefly describe the possible causes of oxidative stress in obesity, and the impact of obesity-induced oxidative stress on adipose tissue function. We also summarize the crosstalk between adipose tissue and vasculature mediated by adipocytokines in vascular oxidative stress. In addition, we highlight the potential target mediating adipose tissue oxidative stress.

## Introduction

The prevalence of obesity has been rapidly growing in the past few decades (1). Obesity is defined as abnormal or excessive accumulation of fat, which may impair health (2). Obesity is a major risk factor for type 2 diabetes mellitus, cardiovascular diseases (CVDs), and several cancers (3). These diseases are together known as noncommunicable diseases, accounting for over 70% of the early deaths globally and ranking the leading cause of mortality and premature disability (4). Obesity is assessed by body mass index (BMI), which is the ratio of body weight and height (kg/m^2^). Persons with a BMI equal or over 30 kg/m^2^ are diagnosed as obesity (2). Elevated BMI accounted for 4 million deaths worldwide in 2015, more than two thirds of which were caused by CVDs (5).

The excessive fat accumulation in obesity is primarily caused by overconsumption of calories and lack of physical activities (6). Adipose tissue, as a caloric reservoir, expands to accommodate overnutrition, which leads to the dysfunctional remodeling (7). Bioactive substances secreted from adipose tissue, known as adipokines, exert important functions in regulating systemic metabolism and inflammation. The obesity-induced adipose tissue dysfunction affects the adipokines secretion profiles, which further regulates remote tissues including cardiovascular systems (8). In addition, the expansion of adipose tissues also causes ectopic fat deposition in other organs, such as liver, heart, and kidney, further exacerbating metabolic disorders (7).

**Figure 1:**
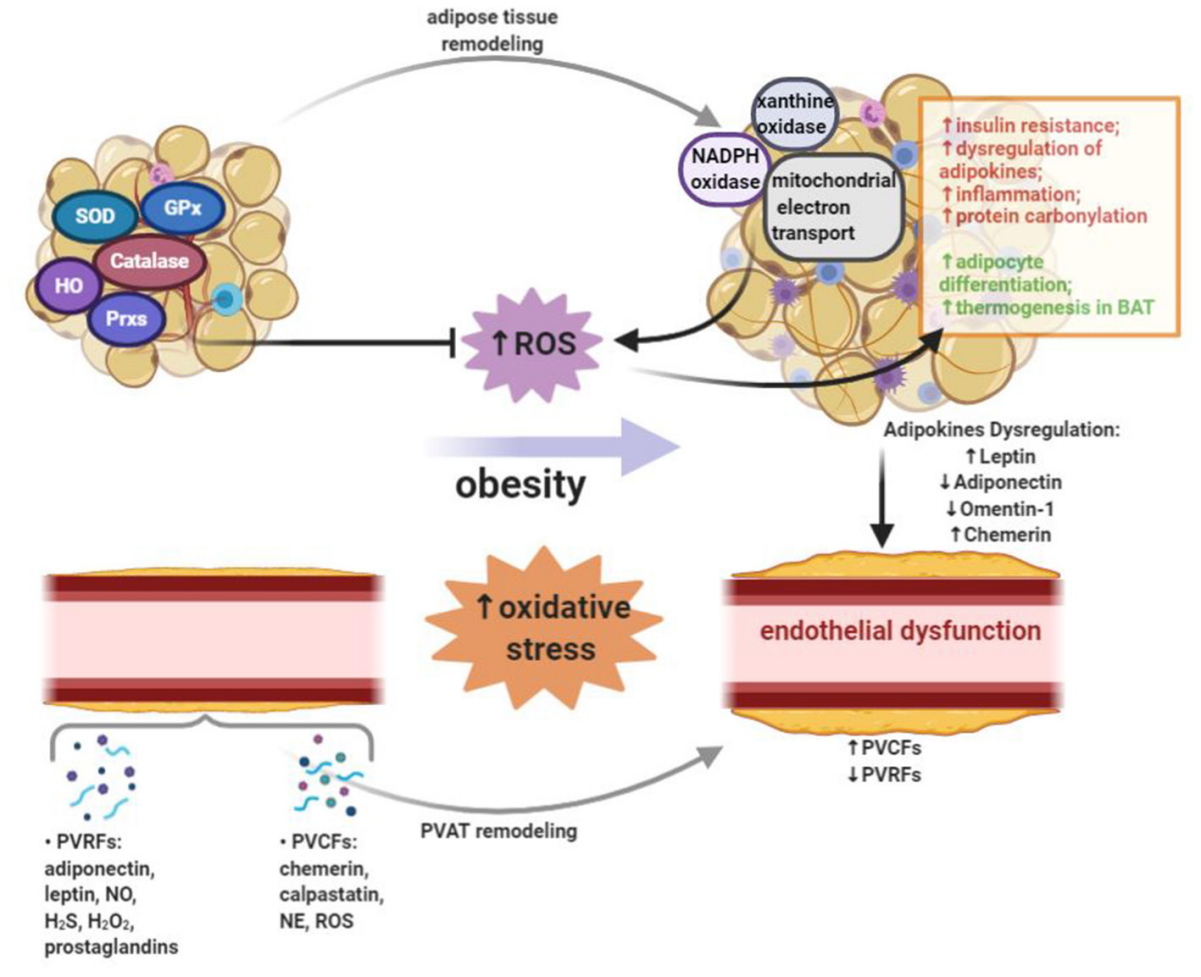
Oxidative stress occurs when the production of reactive oxygen species (ROS) exceeds the antioxidant defense. Obesity leads to the increased systemic oxidative stress. In adipose tissue, ROS can be generated by NADPH oxidase, xanthine oxidase, and mitochondrial oxidative phosphorylation system. On one hand, the production of ROS in adipose tissue of obese subjects leads to insulin resistance, dysregulated adipokines secretion, inflammation and increased protein carbonylation. On the other hand, ROS in adipose tissue could promote adipocyte differentiation and thermogenesis in brown adipose tissue (BAT). A variety of enzymes, including superoxide dismutase (SOD), catalase, glutathione peroxidases (GPx), heme oxygenase (HO), and peroxiredoxins (Prxs), can reduce ROS burden and act as antioxidant defense in adipose tissue. Adipose tissue exerts direct effects on vascular system through releasing a wide range of bioactive products, which include circulating adipokines. Perivascular adipose tissue (PVAT) is important adipose tissue that regulates vascular function and remodeling due to its close proximity. In PVAT, the modulation of vascular contractility is conducted through the secretion of PVAT-derived relaxing factors (PVRFs) and PVAT-derived contracting factors (PVCFs). In obesity, increased oxidative stress, inflammation and eNOS dysfunction in PVAT may alter the balance between PVRFs and PVCFs. Obesity-induced PVAT dysfunction leads to the reduction of PVRFs and the production of PVCFs, hence causing enhanced vasocontraction. In addition, chronic changes in the adipokines profile may result in the pathological vascular remodeling which can further increase the risk of CVDs.

Oxidative stress is accompanied with both obesity and CVDs. Oxidative stress occurs when the production of reactive oxygen species (ROS) exceeds the antioxidant defense (9). Normally, ROS are involved in homeostatic signaling and are secondary messengers in various important intracellular signaling pathways (10). The abnormal generation of ROS and oxidative stress in adipose tissue emerge as a potential pathophysiological mechanism underlying vascular dysfunction. For example, the important anticontractile function of perivascular adipose tissue (PVAT) is lost after obesity-induced oxidative stress (11). Therefore, oxidative stress in adipose tissues could be targeted for the prevention and treatment of vascular dysfunction. In this review, we summarize current understanding of how oxidative stress induced by obesity contributes to adipose tissue dysfunction, and further promotes the development of vascular dysfunction (Figure 1).

## Oxidative Stress in Obesity

### Oxidative Stress and ROS

ROS are generated during aerobic metabolism of oxygen. The primary sources of ROS include the mitochondrial respiratory chain and oxidase enzymes, such as nicotinamide adenine dinucleotide phosphate (NADPH) oxidases, xanthine oxidase (XO), lipoxygenases, cyclooxygenases, cytochrome P450 enzymes and uncoupled nitric oxide synthases (12). Under normal conditions, small quantities of ROS, such as superoxide (${\text{O}}_{2^{\;-}}$), hydrogen peroxide (H_2_O_2_), hydroxyl radical (^·^OH) and peroxynitrite (ONOO^−^), are neutralized by the antioxidant defense systems. Under the pathological conditions, however, enzymatic production of ROS exceeds the available antioxidant defense systems, leading to the state of oxidative stress (13). The accumulated ROS cause cellular dysfunction and tissue damage by direct oxidative modification of biomolecules, and modulate redox-sensitive signal transduction pathways and transcription factors (14). For instance, the reaction between ${\text{O}}_{2^{\;-}}$ and the gaseous messenger nitric oxide (NO) leads to the deprivation of NO bioactivity, while the reaction product ONOO^−^ causes endothelial nitric oxide synthase (eNOS) dysfunction and hence reduced NO production (13).

### Anatomical Features of Adipose Tissue

In human body, adipose tissue can be commonly classified as subcutaneous adipose tissue (SAT) and visceral adipose tissue (VAT) by their anatomical localizations (15). It is now widely recognized that expansion of SAT has a minor contribution to or even a positive impact on cardiometabolic diseases, while expansion of VAT is significantly associated with increased cardiometabolic risks (16). Recently, it is suggested that obesity cannot be evaluated simply by BMI, because the amount of VAT and ectopic fat also significantly defines the risk of developing CVDs in overweight and moderate obese subjects (17). Although cumulative weight burden tends to aggravate arterial diseases in children and adults (18, 19), visceral fat volume has been shown to be more associated with systemic endothelial dysfunction than subcutaneous fat (20, 21). Therefore, numerous clinical studies have been using adiposity measures, including waist circumference and waist to hip ratio, to indicate central obesity (22).

Obesity-induced oxidative stress in adipose tissue is regulated in a depot-specific manner. Epicardial adipose tissue, adjacent to coronary arteries and myocardium, produced a higher level of ROS than SAT in patients with CVDs (23). Oxidized lipids and proteins tended to accumulate in VAT compared to SAT in both obese mice and humans (24, 25). The level of aldehyde products of lipid peroxidation was increased in epididymal adipose tissue but decreased in SAT in both high-fat diet-induced obese mice and *ob/ob* mice (26). Furthermore, exercise training-mediated oxidative stress reduction, measured by decreased level of lipid peroxidation and NADPH oxidase expression, was more remarkable in VAT than in SAT (27).

Based on function and gene expression profile, adipose tissue can be further categorized into white, brown, and beige adipose tissue. White adipose tissue (WAT), including SAT and VAT, represents most of the fat mass in humans. Brown adipose tissue (BAT) accounts for around 4.3% of overall fat mass in adults and is located predominantly in the interscapular and supraclavicular region (28). BAT is highly vascularized and consists mostly brown adipocytes. Morphologically, brown adipocytes have numerous small lipid droplets and a much higher number of mitochondria, which leads to their brown color and multilocular histological appearance (29). Upon cold exposure or certain pharmacological stimulations, beige adipocytes can be induced in WAT depots (30, 31). Beige adipocytes, similar to brown adipocytes, exhibit multilocular lipid droplets and are packed with a high number of mitochondria (32). BAT and beige adipose tissue activations are linked to non-shivering thermogenesis (28), but more importantly, their activations have been shown to promote energy expenditure and improve insulin sensitivity (33).

In the past years, PVAT has been identified as an important regulator of vascular functions due to its proximity to the vasculature (34). For a long time, PVAT was thought to be a connective tissue providing mechanical protection to the surrounding vessels (35). However, with the reveal of the endocrine and paracrine functions of PVAT, it has been recognized that PVAT could regulate vascular tone through secreting adipokines, chemokines, and hormone-like factors (36). The anticontractile function of PVAT has been demonstrated in both rodents and humans (11, 37). Several studies indicated that PVAT in different anatomic locations could resemble different adipose tissues (32). In rodents, abdominal PVAT and mesenteric PVAT are morphologically more like WAT, whereas the thoracic periaortic adipose tissue resembles BAT from a morphological and functional standpoint (38).

### Increased Systemic Oxidative Stress in Obesity

It has been shown that obesity contributes to the development of systemic oxidative stress in both human and animal studies (39, 40). Systemic oxidative stress was positively correlated with fat mass in humans and rodents (39). Mitochondrial oxidative stress markers, such as protein carbonyls, lipid peroxidation products, and malondialdehyde (MDA), as well as ROS production were increased in adipose tissues of obese subjects (41). In *db/db* mice, ROS production was upregulated in adipocytes, along with lipid peroxidation product 4-hydroxynonenal (4-HNE) accumulation (42).

Possible triggers of obesity-induced oxidative stress include altered nutritional status, hyperglycemia, hyperlipidemia, and chronic inflammation (43). Consumption of high-fat, high-carbohydrates food induced prolonged oxidative stress and inflammation in obese people (44). Moreover, lack of dietary consumption of protective antioxidant phytochemicals caused decreased plasma levels of vitamins and minerals in overweight and obese subjects, hence contributing to increased oxidative stress (45–47).

Obesity is closely related to hyperglycemia and insulin resistance. A strong induction of ROS accumulation in cultured adipocytes was associated with exposure of the cells to hyperglycemia condition, while *in vivo* study, with streptozotocin-treated hyperglycemic mice, showed an enhanced oxidative damage to DNA in adipose tissue (48). Obesity is also accompanied with increased circulating free fatty acids (FFA). FFA, such as palmitate, stimulate ROS production via protein kinase C (PKC)-dependent activation of NADPH oxidase in smooth muscle cells and endothelial cells (49). In addition to FFA, the high cytosolic triglyceride level in obesity correlates to elevated levels of cytosolic long-chain acyl-CoA esters, which can inhibit mitochondrial adenine nucleotide translocators and lead to an intramitochondrial adenosine diphosphate (ADP) deficiency. The ADP deficiency, *in vitro*, is a strong promoter of mitochondrial ROS production (50).

Low-grade chronic inflammation is an important cause of oxidative stress in obesity (51). Although adipocytes consist mostly of the adipose tissue volume, adipose tissue contains another heterogeneous cell population, referred to as stromal vascular fractions (SVFs). SVFs include preadipocytes, fibroblasts, vascular endothelial cells, and immune cells. Accompanied with adipose tissue expansion, obesity leads to a quantitative and qualitative alteration in cellular composition in adipose tissue. Among all the immune cells in adipose tissue, macrophages are the most abundant immune cells in obese subjects. The recruitment and proliferation of macrophages under high-fat diet condition are directly linked to adipose tissue inflammation. There are two distinct population of macrophages, the pro-inflammatory M1 macrophages and the anti-inflammatory M2 macrophages. The pro-inflammatory cytokines, such as tumor necrosis factor-alpha (TNF-α), interleukin (IL)-6, and IL-1β, secreted from activated M1 macrophages increase ROS production in adipose tissue of obese individuals (52). Treatment of 3T3-L1 adipocytes with TNF-α decreased the expression of mitochondrial antioxidants, including glutathione S-transferase A4 (Gsta4), peroxiredoxin 3 (Prx3), and glutathione peroxidases (GPx), therefore resulting in increased protein carbonylation, ROS generation, and mitochondrial dysfunction (26, 53). Mice with a myeloid cell-specific NADPH oxidase 2 (Nox2) knockout showed protective effect on high-fat diet induced adipose tissue inflammation and improved metabolic functions (54).

## The Role of Oxidative Stress in Adipose Tissue Function

### Sources of ROS in Adipose Tissue

Several enzyme systems have been identified that can generate ROS in adipocytes. In adipose tissue, ROS can be produced by NADPH oxidase (Nox), XO, and the mitochondrial oxidative phosphorylation system. In the time course of obesity, sources of ROS in adipose tissue may change from Nox4 primarily in adipocytes at early stage, to Nox2 in macrophages at intermediate stage, and finally to mitochondria oxidative phosphorylation at later stage (55).

Nox is a multicomponent enzyme, which produces ROS when transferring electrons from NADPH across the cell membrane to oxygen. The Nox family consists of seven isoforms, including Nox1, Nox2, Nox3, Nox4, Nox5, Duo1, and Duo2 (56). Among the Nox family members, Nox4 is the only isoform expressed in adipocytes (57). During obesity development, induced by a high-fat and high-sucrose diet, adipocyte Nox4 and pentose phosphate pathway activity were transiently increased in mice (58). Mice with adipocyte-specific Nox4 deficiency were protected against obesity-induced insulin resistance (58). Glucose and palmitate induced ROS production in adipocytes through activation of Nox4 rather than mitochondrial oxidation (57). Primary adipocytes isolated from Nox4 knockout mice were resistant against high glucose or palmitate-induced inflammation (58). In cultured adipocytes, fatty acids treatment promoted oxidative stress through activation of Nox (39). In obese mice, the expressions of Nox subunits, gp91phox, p47phox, p22phox, p67phox, were upregulated in WAT (39). In addition, treatment with Nox inhibitor reduced ROS production in WAT of obese mouse models, and improved obesity-induced adipose tissue dysfunction (39).

Mitochondrial source of ROS has been regarded as a major trigger of oxidative stress in adipose tissue. Mitochondria produce energy through oxidative phosphorylation, during which a portion of oxygen molecules generate ROS. Complex I and III of the electron transport chain (ETC) are the main sites of ROS production. In complex I, electrons are removed from nicotinamide adenine dinucleotide (NADH) or the reverse reaction of complex II occurs, which leads to premature electron leakage to oxygen and ROS production in mitochondrial matrix. In complex III, ROS are produced in both sides of mitochondrial inner membrane (59). In obesity, the excessive nutrients accumulated in adipocytes increase mitochondrial substrate loading, resulting in an enhanced ROS generation in mitochondria (60). *In vitro* studies showed that high concentration of glucose or FFA increased ROS generation in mitochondria (48, 61, 62).

Another enzyme system linked to adipocyte ROS generation is the xanthine dehydrogenase (XDH)/oxidoreductase (XOD) system. Under physiological conditions, XOD dominates in the form of XDH, whereas under oxidative stress, XOD converts into XO, which is considered as the major source of ROS production (63). XO is an oxidant form of XOD, which converts purine bases to uric acid (63). In a clinical study with overweight and obese volunteers, obesity was an independent predictor of high XO activity (63). Besides, obesity is often accompanied by hyperuricemia (64). The production of uric acid through XOD was enhanced in adipose tissue of obese mice (64). The process of purine catabolism, conversion of xanthine to uric acid, generates ROS, such as ${\text{O}}_{2^{\;-}}$ and H_2_O_2_, which potentially links the *de novo* lipogenesis with ROS production (52).

### Effect of ROS in Adipose Tissue

Obesity-induced oxidative stress in adipose tissue is a major contributor to cellular dysfunction and insulin resistance (65). Prolonged exposure of 3T3-L1 adipocytes to H_2_O_2_ resulted in an impaired insulin-induced activation of glucose transporter type 4 (GLUT4) (66). In obesity, the increased ROS production is closely related to the dysregulation of adipokines expression in adipose tissues (67). Oxidative stress in adipose tissue activated nuclear factor kappa-light-chain-enhancer of activated B cells (NF-κB) and mitogen-activated protein kinase (MAPK), which further downregulated anti-inflammatory adipokines and upregulated pro-inflammatory cytokines in adipose tissue (67). In cultured adipocytes, increased oxidative stress led to dysregulation of adipocytokines production, including adiponectin, IL-6 and monocyte chemotactic protein 1 (MCP1) (39).

Protein carbonylation, the irreversible modification of proteins by reactive lipid aldehydes, is a major result of oxidative stress in adipose tissue. The most widely studied aldehyde products of lipid peroxidation are 4-HNE and 4-oxononenal (4-ONE), which are abundantly present in adipose tissue (26). In obese mouse models, levels of lipid peroxidation products were increased 5 to 11-fold in epididymal adipose tissues (26). Carbonylation of histones by 4-HNE was potentiated in adipose tissue of *ob/ob* mice and high-fat diet-induced obese mice (68).

Although ROS are often correlated with CVDs and negative metabolic outcome, they also play important regulatory roles in adipose tissue biology. 3T3-L1 cell differentiation was accelerated after H_2_O_2_ treatment, which was mediated by upregulation of peroxisome proliferator-activated receptor gamma (PPARγ) expression (61). Acute activation of thermogenesis in BAT induced a substantial increase of mitochondrial ROS, whereas pharmacological depletion of mitochondrial ROS resulted in hypothermia upon cold exposure and inhibited uncoupling protein 1 (UCP1)-dependent enhancement in whole body energy expenditure (69).

### Antioxidant Systems in Adipose Tissue

There are a variety of enzymes that can reduce ROS burden and act as antioxidant defence. These enzymes include superoxide dismutase (SOD), catalase, heme oxygenase (HO), peroxiredoxins (Prxs), and GPx (13).

Catalase, mainly present in peroxisomes, is a cellular antioxidant enzyme that eliminates excessive H_2_O_2_. Attenuating ROS emission, either by treatment of antioxidant or by genetically overexpression of catalase, has been shown to improve obesity-induced metabolic disorders (70, 71). Catalase-knockout mice exhibited more weight gain and higher fat mass under either normal chow or high-fat diet feeding conditions than control mice (72, 73). This phenotype of catalase-knockout mice can be attenuated by concomitant treatment with antioxidant, melatonin or N-acetyl cysteine (73). *In vitro* study, using 3T3-L1-derived adipocytes transfected with catalase-small interfering RNA, revealed an increased lipogenesis and Nox4 expression in catalase-deficient cells (73).

HO is an enzyme catalyzing the degradation of heme that is a pro-oxidant and strong inducer of HO1, thereby producing biliverdin, ferrous iron and carbon monoxide. While HO1 is the stress inducible isoform, HO2 is the constitutive isoform that is expressed under homeostatic conditions. Adipocyte-specific HO1 knockout caused an enhanced fasting hyperglycemia and insulinemia in female mice, but not male mice on both standard diet and high-fat diet, indicating a greater protective role of HO1 in females (74). Lentiviral-mediated adipocyte-specific overexpression of human HO1 in mice led to an increased human HO1 expression in adipose tissue without affecting endogenous murine HO1 (75). The adipocyte-specific overexpression of HO1 attenuated high-fat diet induced adiposity and vascular dysfunction and improved insulin sensitivity and adipocyte function through modulating adiponectin level and inflammation (75).

Prxs, a ubiquitous family of antioxidant enzymes, control the cytokine-induced peroxide levels in mammalian cells. Prxs can be regulated by phosphorylation, oxidation, reduction or oligomerization (76). Prxs family members are classified by their intracellular location. Prx2, present in the cytoplasm and cell membranes, is significantly involved in intracellular redox balance with its ROS scavenging activity (77). Prx2 expression was upregulated during adipocyte differentiation. Silencing of Prx2 in 3T3-L1 cells increased ROS production and inhibited adipogenesis via modulating adipogenic gene expression, which suggested that Prx2 deficiency caused adipocyte dysfunction and cell death via promoting ROS production (78). The mitochondrial antioxidant Prx3 decreased significantly in adipose tissue of obese mice and humans, contributing to a state of oxidative stress in obesity (79). Inflammation induced by obesity is associated with decreased expression of mitochondrial antioxidant genes in epididymal adipose tissue, such as Prx3, Gsta4, aldehyde dehydrogenase 1 (Aldh1), and GPx-4 (71). Prx3 knockout mice exhibited adipocyte hypertrophy and increased mitochondrial protein carbonylation (79). 3T3-L1 cells with Prx3 knockdown showed increased ROS production, decreased mitochondrial potential, and lower adiponectin expression (79).

Mice, with adipocytes overexpression of antioxidant enzyme, catalase and SOD1, showed adipose expansion with decreased ectopic fat accumulation and improved insulin sensitivity (80). On the contrary, mice with glutamate-cysteine ligase (Gclc) deleted in adipocytes, exhibited restricted adipose tissue expansion associated with increased ectopic fat accumulation and deteriorated insulin sensitivity (80). In this Gclc adipocyte-specific knockout mice, glutathione synthesis was disabled, and ROS production was increased in adipocytes. *In vitro* studies revealed that oxidative stress resulted in suppression of *de novo* lipogenesis, possibly through lysine K-specific demethylase 1A (KDM1A)-mediated attenuation of sterol-regulatory element-binding transcription factor 1 (SRBF1) transcriptional activities (80).

**Figure 2:**
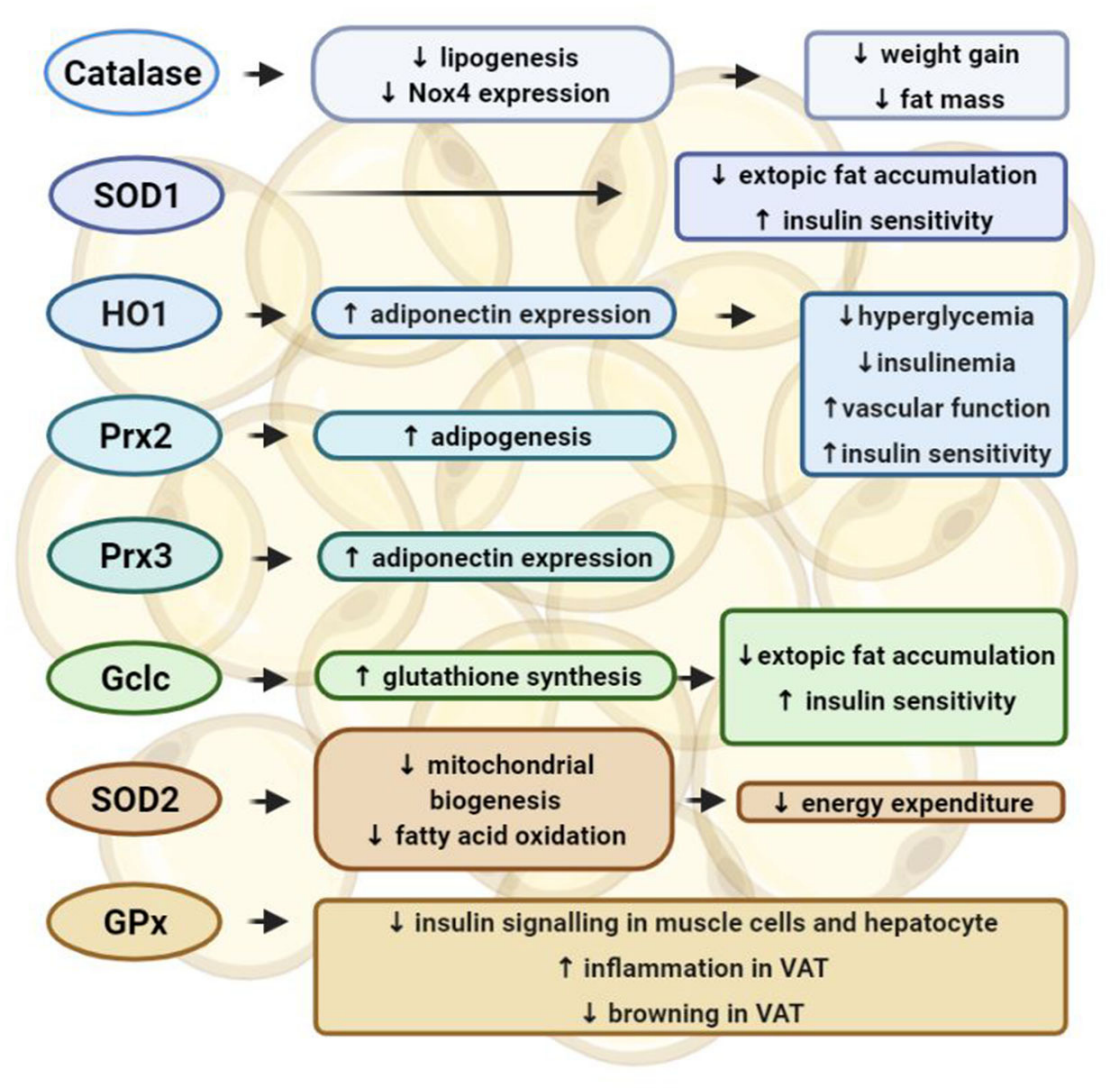
Functions of the antioxidant enzyme systems in adipose tissues. In adipocytes, catalase inhibits lipogenesis and Nox4 expression, which prevents weight gain and fat mass increase induced by high-fat diet. Overexpression of catalase and superoxide dismutase (SOD)-1 in adipocytes can inhibit ectopic fat accumulation and improve insulin sensitivity. Heme oxygenase 1 (HO1) can stimulate the expression of adiponectin, therefore preventing hyperglycemia and insulinemia in female mice, and improving vascular function and insulin sensitivity. Peroxiredoxin (Prx)-2 can promote adipogenesis, whereas Prx3 can stimulate the expression of adiponectin. Depletion of these antioxidant enzymes in adipocytes has shown detrimental effects in adipocyte functions and promoted cardiometabolic diseases. Glutamate-cysteine ligase (Gclc) facilitates glutathione synthesis and inhibits ROS production, thereby inhibiting ectopic fat accumulation and insulin resistance. On the other hand, deletion of either SOD2 or glutathione peroxidases (GPx) has been reported to provide beneficial effect in adipose tissue function. The anti-obesity effect of SOD2 deletion in adipocytes can be attributed to an activated mitochondrial biogenesis and enhanced mitochondrial fatty acid oxidation, which can promote energy expenditure. Insulin signaling can be enhanced by knocking down GPx in either muscle cells and hepatocytes. GPx-1 deletion can attenuate inflammation and enhance browning in visceral adipose tissues.

Whereas, these data suggest that increasing mitochondrial antioxidants protects against oxidative stress in adipose tissue, other studies also reveal different phenotypes. SOD catalyzes the dismutation of ${\text{O}}_{2^{\;-}}$ into oxygen and H_2_O_2_, serving as a key antioxidant. However, mice with an adipocyte-selective knockout of SOD2, the isoform in mitochondrial matrix, exhibited resistance to obesity induced by high-fat diet and enhanced energy expenditure (81). The anti-obesity effect of SOD2 deletion in adipocytes was attributed to an activated mitochondrial biogenesis and enhanced mitochondrial fatty acid oxidation (81). GPx is an antioxidant enzyme family with peroxidase activity, which converts lipid hydroperoxides to their corresponding alcohols and free H_2_O_2_ to water. However, mice lacking GPx-1 were protected from high-fat diet-induced insulin resistance, which was due to an improved insulin signaling in muscle cells (82). Mice with hepatocyte-specific deficiency in GPx-1 showed an enhanced hepatic insulin sensitivity and were protected from diet-induced non-alcoholic steatohepatitis (83). High-fat diet-induced glucose intolerance and hepatic steatosis were improved in mice with both GPx-1 and catalase knockout, which was due to attenuated inflammation and enhanced browning in visceral adipose tissues (84) (Figure 2).

## The Interplay of Adipose Tissue and Vasculature

### Adipokines in Vascular Oxidative Stress

Adipose tissue exerts direct effects on vascular systems through releasing a wide range of bioactive products, including adipokines [leptin (85), adiponectin (86, 87), omentin-1 (88) and chemerin (89)], inflammatory cytokines and chemokines (90), gaseous messengers such as hydrogen sulfide (H_2_S) (91) and NO (92), ROS (93), microRNAs (94), microvesicles (95), and fatty acid metabolites (96).

In the vasculature, these bioactive molecules are involved in regulation of (1) local redox state through alteration of Nox activity and eNOS coupling; (2) endothelial function through modulation of eNOS activity and NO production; (3) inflammation; (4) neointima formation; (5) vascular smooth muscle cell migration; (6) local endothelial cell activation (97). On the contrary, changes in vascular biology, such as increased oxidative stress and inflammation, can also affect the functions of adipose tissue, particularly PVAT, in a bidirectional loop. Lipid peroxidation products, such as 4-HNE, and inflammatory cytokines produced in vascular wall can diffuse to the surrounding PVAT and activate specific signaling pathways in adipocytes (98, 99).

#### Leptin

Leptin is best known for its interaction with central nervous system to decrease food intake and increase energy expenditure (100). However, leptin also plays an important role in endothelial functions. Obesity is associated with the increased circulating leptin level, which is an independent risk factor for a number of vascular diseases (101). Leptin was also shown to increase arterial pressure when infused chronically in rodents (102). In human umbilical vein endothelial cells (HUVEC), leptin increased the production of ROS and the expression of MCP1, indicating that chronic oxidative stress in endothelial cells, induced by hyperleptinemia, may contribute to vascular dysfunction (103). Another study carried in bovine aortic endothelial cells showed that leptin increased ROS generation by promoting fatty acid oxidation through protein kinase A activation (104). In HUVEC, exposure to leptin induced eNOS expression and reduced intracellular L-arginine, leading to eNOS uncoupling accompanied with reduced NO bioavailability and increased cytotoxic ONOO^−^ (105). In human aortic endothelial cells, leptin-induced eNOS activation was mediated through AMP-activated protein kinase (AMPK)/Akt signaling pathways, which could be blunted by the interaction between leptin and C-reactive protein (CRP) (106). Besides, leptin may directly mediate vascular tone, since leptin at a high concentration caused endothelium-dependent NO-mediated vascular relaxation in vessels from control, but not obese rats (101).

#### Adiponectin

Adiponectin, an adipokine involved in diabetes and insulin resistance, appears to be the link between obesity and CVDs (107). Adiponectin produced in PVAT exerts paracrine effects on the vascular wall (11). In patients undergoing coronary artery bypass graft surgery, circulating adiponectin level was independently associated with NO bioavailability and ${\text{O}}_{2^{\;-}}$ formation/eNOS uncoupling in saphenous vein segments and internal mammary arteries. However, vascular ${\text{O}}_{2^{\;-}}$ formation and eNOS uncoupling were positively correlated with adiponectin gene expression/release in PVAT (87). Adiponectin improved eNOS coupling in the underlying vessels, via induction of Akt-mediated eNOS phosphorylation and increasing tetrahydrobiopterin (BH_4_) bioavailability (87). *Ex vivo* experiments showed that peroxidation products, such as 4-HNE, produced in the vascular wall downregulated adiponectin gene expression in PVAT through a PPARγ-dependent mechanism (87). Analysis of internal mesenteric artery (MA) and their adjacent PVAT, revealed a significant correlation of type 2 diabetes with both hypoadiponectinemia and increased vascular ${\text{O}}_{2^{\;-}}$ production (108).

The conserved paralog of adiponectin, complement C1q/tumor necrosis factor-related protein (CTRP) has been shown to exert protective effects in the cardiovascular system (109). In diet-induced obese mice, treatment of CTRP-9 promoted phosphorylation of eNOS and reduced ${\text{O}}_{2^{\;-}}$ production and TNF-α levels in PVAT, which further improved anticontractile effect of PVAT (110). Adiponectin and CTRP-9 exert beneficial effects on vascular function through improving eNOS coupling. Although circulating adiponectin level was reduced in type 2 diabetic patients, adiponectin expression in PVAT was increased and positively correlated with vascular ${\text{O}}_{2^{\;-}}$ production (108). Additionally, treatment of human arteries with recombinant adiponectin directly reduced the production of ${\text{O}}_{2^{\;-}}$ by Nox (108). In high-fat diet-fed obese mice, increased oxidative stress in PVAT mediated by TNF-α, resulted in loss of PVAT anticontractile effect (111).

#### Omentin-1

Omentin-1, also known as intelectin-1, is an anti-inflammatory adipokine which is produced primarily by SVFs within adipose tissue. In diabetic Goto-Kakizaki rats fed with or without high-fat diet, vascular ${\text{O}}_{2^{\;-}}$ production was decreased and NO bioavailability was improved by omentin-1 treatment (88). Besides, omentin-1 ameliorated endothelium dysfunction in the obese diabetic rats by improving endothelium-dependent relaxation to acetylcholine (88).

#### Chemerin

Whereas, adiponectin and CTRP9, exert beneficial effect on vascular function through improving eNOS coupling, the adipokine chemerin has an opposing effect, that is to promote eNOS uncoupling and reduce NO production in favor of ROS (89). Chemerin has been identified as an endogenous vasoconstrictor, and its expression is upregulated in hypertensive rats (112). TNF-α can stimulate chemerin production from adipocytes, which indicates the linkage between inflammation and chemerin secretion (113).

### PVAT Oxidative Stress in Obesity-Related Vascular Dysfunction

The crosstalk between PVAT and the vasculature is important for vascular function. PVAT exerts its anticontractile function through secretion of PVAT-derived relaxing factors (PVRFs), which include adiponectin, leptin, NO, H_2_S, H_2_O_2_, and prostaglandins (114, 115). In addition to PVRFs, PVAT-derived contracting factors (PVCFs) have been recently identified. These PVCFs, including chemerin, calpastatin, norepinephrine (NE) and ROS, can modulate vasoconstriction (116–118).

PVRFs regulate vascular tone in both endothelium-dependent and -independent manners (119). In endothelium-dependent mechanism, PVRFs modulate NO production and the activation of calcium-dependent potassium channels to facilitate vasorelaxation. On the other hand, the endothelial-independent anticontractile property of PVAT may involve the generation of H_2_O_2_ and subsequent guanylyl cyclase activation (119). PVAT ROS are continuously generated from the mitochondria in response to contractile stimuli. ROS, as important mediators of PVAT anticontractile effects, act directly on VSMC (120).

In obesity, PVAT dysfunction leads to the dysregulation of PVAT-derived vasoactive factors, which in turn affects the vascular function. The anticontractile responses to vasodilators showed no significant differences between obese and lean mice if the PVAT were removed from the vessels, suggesting that obesity did not directly impair the intrinsic vascular function, but rather the function of PVAT (121). The shift toward a proinflammatory and prooxidative state of PVAT may lead to endothelial dysfunction (122). Under normal conditions, the deleterious effects of ROS are neutralized by the antioxidant enzymes in PVAT (123, 124), whereas in obesity, the increased oxidative stress and inflammatory response, together with the dysfunction of eNOS and NO result in PVAT dysfunction (125).

In rats, MA with intact PVAT showed a greater contraction to perivascular nerve stimulation than those without PVAT, which was mediated via the production of ROS by Nox in PVAT (123). MA incubated with aortic PVAT from high-fat diet-fed rats showed reduced endothelium-dependent relaxation compared to those incubated with PVAT from normal chow diet-fed rats (126). High-fat diet significantly increased the mass of PVAT and the number of hypertrophic adipocytes, and promoted the shift to a WAT characteristic of PVAT in rodents (126). In diet-induced obese mice, abdominal aortic PVAT showed increased formation of ROS, including H_2_O_2_ and ${\text{O}}_{2^{\;-}}$ (127). Moreover, high-fat diet-fed mice showed reduced expression of SOD3 and glutathione levels in mesenteric PVAT (128). Abdominal aorta with PVAT displayed impaired endothelium-dependent vasodilation, whereas the vasodilation was restored after inhibition of ROS formation in vascular wall or simply removal of PVAT (127). Obesity-induced oxidative stress in PVAT was exacerbated in aged mice, leading to vascular oxidative stress and inflammation (129). Inflammatory factors secreted from PVAT of obese aged mice significantly promoted prooxidative and proinflammatory phenotype in cultured arteries isolated from young healthy mice (129).

### BAT Oxidative Stress in Obesity-Related Vascular Dysfunction

BAT-specific adipokines, referred to as batokines, exert beneficial endocrine, paracrine and autocrine effects on peripheral tissues, including vascular systems (31). In high-fat diet-induced obesity, BAT showed higher levels of ROS generation and enhanced antioxidant enzyme activity compared with lean counterparts (130). Large amount of ROS, such as ${\text{O}}_{2^{\;-}}$, H_2_O_2_, and oxidized lipids are generated in BAT upon acute activation of thermogenesis (69, 131). Mitochondrial ROS accumulated in BAT converge on UCP1 C253, inducing cysteine sulfenylation (69). UCP1 C253A desensitizes UCP1 to adrenergic activation, however, it does not impair the thermogenesis in brown adipocytes (69). The anticontractile effect of BAT on vasculature was abolished in the Nox4 knockout mice. BAT could induce the activation of cyclic GMP-dependent protein kinase G type-1α (PKG-1α) through Nox4-derived H_2_O_2_, leading to a reduced vascular contractility (93). PVAT from β3 agonist-treated mice exhibited a browning phenotype and an increased anti-contractile effect (93).

Currently, there are a few well-known strategies that can induce browning in adipose tissues, which include cold acclimation and treatment of growth factors such as fibroblast growth factor 21 (FGF21) (132), atrial natriuretic peptide (ANP) (133), and bone morphogenetic protein (BMP) (134). Cold acclimation can stimulate triglyceride clearance and glucose uptake in adipose tissues, which contribute to the modulation of oxidative stress (135, 136). In mice, cold acclimation inhibited high-fat diet-induced endothelial dysfunction and atherosclerosis, which were associated with a significant reduction in levels of proinflammatory markers (137). In high-fat diet-fed rats, cold acclimation stimulated the browning of abdominal aortic PVAT, which was accompanied by the increased expression of phospho-AMPK, UCP1 and peroxisome proliferator-activated receptor gamma coactivator 1-alpha (PGC-1α), as well as the reduced expression of TNF-α, IL-6, and p65 (138).

Apart from cold exposure, a well-recognized antioxidant melatonin is also an activator of BAT. Melatonin, a derivative of tryptophan, is secreted primarily during the dark phase of the light/dark cycle by pinealocytes. It is regarded as an important chronobiotic regulating internal biological clock (139). In addition, melatonin is also involved in energy metabolism (140, 141). Melatonin supplementation prevented rodents against obesity without affecting food intake and physical activities (142–144). In fact, melatonin can increase energy expenditure through activation of BAT and preserve mitochondrial functions (145, 146). Clinically, melatonin also plays an important role in maintaining cardiovascular homeostasis through regulating blood pressure and is regarded as a putative antihypertensive treatment (147). Melatonin may exert its cardiovascular protective effect through its free radical scavenger activity and indirectly through its activation of BAT.

## Potential Targets Mediating Oxidative Stress in Adipose Tissues

### Nrf2

Nuclear factor E2-related factor 2 (Nrf2), a member of the Cap-n-Collar subfamily of basic leucine zipper (bZIP) transcription factors, regulates the expression of antioxidant proteins that protect against oxidative stress. Under normal conditions, Nrf2 is kept in the cytoplasm by Kelch like-ECH-associated protein 1 (Keap1) and Cullin 3, which degrade Nrf2 by ubiquitination. Oxidative stress disrupts the Keap1-Cullin 3 ubiquitination system, leading to Nrf2 translocation to the nucleus. In the nucleus, Nrf2 binds to the antioxidant response element (ARE) in the upstream promoter region of many antioxidant genes and initiates the transcription, including NADPH quinone oxidoreductase 1 (NQO1) and HO1 (148). In obese mouse models and human subjects, oxidative stress was significantly increased in WAT (149). The oxidative stress in WAT induced Nrf2 expression and activity, further exacerbating lipid accumulation in adipocytes and promoting the development of obesity (149). Oxidative stress-induced lipid accumulation in WAT was reduced in Nrf2 knockout mice (149). Mechanistically, Nrf2 mediated lipogenesis through binding to sterol regulatory element binding protein 1 (SREBP1) promoter and inducing lipogenic gene transcription. Besides, Nrf2 also participated in lipolysis in adipocytes via modulating protein kinase A (PKA) pathway (149).

### BAMBI

Bone morphogenetic protein and activin membrane-bound inhibitor (BAMBI) is a transmembrane glycoprotein which is highly homologous to type I receptors of the transforming growth factor beta (TGF-β) superfamily. Therefore, BAMBI is regarded as a pseudoreceptor of the TGF-β-related signaling pathway and acts as a negative regulator of the TGF-β signaling pathway (150). Altered BAMBI expression was observed during periods of adipose tissue remodeling (151). BAMBI is a negative regulator of adipogenesis through modulating anti- and pro-adipogenic effects of paracrine factors, such as Wnt-3a, TGF-β1, and BMP (152, 153). A recent study showed that adipocyte-specific depletion of BAMBI caused an induction of Nox4 expression in adipose tissue, thereby promoting ROS generation in cytoplasm and mitochondria. The deficiency of BAMBI in adipose tissue resulted in an enhanced DNA-binding activity of C/EBPβ and promoted adipogenesis (154). These results together suggested that BAMBI may be an important mediator of adipogenesis through regulating ROS. Therefore, manipulation of BAMBI may present a new therapeutic approach to improve adipose tissue function.

### WWP1

WW domain containing E3 ubiquitin protein ligase 1 (WWP1) is a HECT-type ubiquitin E3 ligase that has been implicated in many pathologies. Proteome analysis of 3T3-L1 cells with WWP1 overexpression revealed an increased abundance of several antioxidative proteins and a reduced ROS levels, and *vice versa* (155). In WWP1 knockout mice, oxidative stress markers were increased in WAT after high-fat diet feeding, indicating WWP1 might participate in the antioxidative response (156). However, whole-body glucose metabolism was improved in the obese WWP1 knockout mice compared to wild-type controls (156). Since the mouse model used in this study were not adipocyte-specific knockout, it cannot be ruled out that the deficiency of WWP1 affected insulin sensitivity in other tissues than WAT or the secretion of insulin from pancreatic β-cells.

### Exercise

In rat, exercise training can enhance eNOS expression and reduce oxidative stress in adipose tissues, thereby inducing browning and thermogenic responses (157). In mice, resistance exercise training induced a brown-like adipocyte reprogramming in WAT. The expressions of vascular endothelial growth factor (VEGF), cluster of differentiation 31 (CD31), UCP1, and browning-related genes were increased in adipocytes of mice after 8 weeks of training (158). However, a recent study showed that under thermoneutral conditions, exercise training did not induce browning in obese rats. These rats showed reduced weight gain, but an oxidative signature in the BAT. Proteomics revealed significant changes in 2-oxoglutarate metabolic process, mitochondrial respiratory chain complex IV, carbon metabolism, and oxidative phosphorylation in adipose tissues of obese rats at thermoneutrality. The author suggested a potential exercise-induced UCP1-independent pathway which may modulate the BAT physiology (159).

## Conclusion

Obesity is characterized by the excessive accumulation of fat in adipose tissue, which leads to adipocyte hypertrophy, hypoxia, as well as the development of systemic oxidative stress. Excess calories intake, overloading of mitochondria, and the generation of oxidative stress in adipose tissues lead to adipose tissue dysfunction and insulin resistance. Since adipose tissues, especially PVAT, are responsible for the production of various vasoactive adipokines to modulate vascular function, obesity-induced adipose tissue dysfunction significantly contributes to the pathogenesis of CVDs. Although physiological levels of ROS are required for adipogenic differentiation and act as secondary messengers of the insulin signaling pathway and vasocontraction, excessive ROS have been shown to be a strong risk factor of obesity-related CVDs. Targeting the abnormal ROS production and oxidative stress in adipose tissues emerges as a potential strategy to prevent and treat CVDs. The detailed mechanisms of the two sides of ROS function in adipose tissues should be fully studied. Antioxidant treatments may have negative effect on oxygen consumption and cannot prevent oxidative stress in adipose tissues (160). Therefore, novel and target-specific strategies should be further investigated.

In this review, we have proposed a few potential and novel targets in linking obesity-induced oxidative stress and functions of adipose tissue. Recent results from the Nrf2, WWP1, and BAMBI studies are promising, but further studies using adipocytes-specific knockout mice would provide a more extensive investigation on their roles in adipose tissue. Manipulation of Nrf2, BAMBI and WWP1 may present new therapeutic approaches to improve adipose tissue function. In addition, promoting browning of adipose tissues during the development of obesity might be another crucial strategy to prevent CVDs. The current well-known strategies to trigger browning of adipose tissues include cold acclimation and exercise training. However, the detailed mechanism of browning remains unclear. Future studies could also investigate whether Nrf2, BAMBI, and WWP1 are involved in the browning or thermogenic responses in adipose tissues.

## Author Contributions

YZ wrote the initial draft of the manuscript. HL and NX critically reviewed and edited the manuscript. All authors have read and agreed to the published version of the manuscript.

## Conflict of Interest

The authors declare that the research was conducted in the absence of any commercial or financial relationships that could be construed as a potential conflict of interest.
